# Efficacy of liver transplantation for hilar cholangiocarcinoma: A protocol for a systematic review and meta-analysis

**DOI:** 10.1097/MD.0000000000011626

**Published:** 2018-07-27

**Authors:** Zhaolun Cai, Yuan Yin, Zhaohui Cai, Zhou Zhao, Chaoyong Shen, Xiaonan Yin, Jian Wang, Zhixin Chen, Dan Cao, Bo Zhang

**Affiliations:** a Department of Gastrointestinal Surgery, West China Hospital, Sichuan University, Chengdu, Sichuan; b Jiangsu Province Hospital of TCM, Affiliated Hospital of Nanjing University of TCM, Nanjing; c Department of Medical Oncology, Cancer Center of West China Hospital, Sichuan University, Chengdu, China.

**Keywords:** hilar cholangiocarcinoma, Klatskin tumor, liver transplantation, meta-analysis, systematic review

## Abstract

**Background:**

Either liver transplantation or surgical resection is available for selected patients with hilar cholangiocarcinoma. However, the comparative effectiveness between liver transplantation and liver resection remains unknown. The aim of our study is to evaluate the relative effectiveness between liver transplantation and surgical resection in patients with hilar cholangiocarcinoma.

**Methods:**

We will systematically search for eligible studies in PubMed, Embase, and the Cochrane library. The primary outcomes are overall survival rates including 1-, 3-, or 5-year survival rates. The second outcomes are postoperative complications. The summary results will be pooled using the random-effects model or fixed-effects model according to the heterogeneity of the included studies.

**Results:**

The results will be submitted to a peer-reviewed journal for publication.

**Conclusion:**

This study will provide a comprehensive evidence summary of the comparison between liver transplantation or surgical resection in the treatment of hilar cholangiocarcinoma.

## 1 Introduction

Hilar cholangiocarcinoma (HC) is a rare tumor. Almost 7000 new cases are diagnosed annually in North America, and the incidence of HC is increasing.^[1,2]^ The prognosis of the disease is poor,^[3]^ and a curative treatment remains a formidable challenge because of its aggressive nature and its critical location close to vital structures.^[4,5]^ Surgery is the only potentially curative treatment option for localized HC, and surgical resection with the goal of an R0 resection is the standard of care for selected patients.^[1,6]^ With the development multidisciplinary approach and evolution of liver surgery during the past decades, liver transplantation as a therapeutic option has significantly improved the surgical management of HC.^[1,6]^

Either liver transplantation or surgical resection is available for selected patients with HC. However, the comparative effectiveness between liver transplantation and liver resection for patients with HC remains unknown. In this study, we performed a systematic review and meta-analysis to evaluate the relative effectiveness between liver transplantation and surgical resection in patients with HC.

## 2 Methods

This study will follow PRISMA guidelines^[7]^ and be conducted following an established protocol (PROSPERO: CRD42018067618). Ethical approval is not required because this is a study based on aggregate data and did not involve humans.

### 2.1 Eligibility criteria

The PICOS strategy (patients, intervention, comparisons, outcome, study characteristics) was used to define the eligibility criteria for the study.

#### 2.1.1 Patients and comparison of interventions

Studies which contain patients with HC treated by liver resection and liver transplantation will be included. Studies which provide no sufficient data of survival rates will be excluded. There are no limitations in age, ethnic distribution, and gender.

#### 2.1.2 Outcomes

The primary outcomes are overall survival rates, including 1-, 3-, or 5-year survival rates. The second outcomes are postoperative complications.

#### 2.1.3 Study design

The present study will evaluate published observational studies comparing liver resection and liver transplantation for the treatment of HC.

### 2.2 Information sources

We will systematically search for eligible studies in PubMed, Embase, and the Cochrane library until June 2018. The reference list of relevant studies will be checked to identify additional studies.

### 2.3 Search strategy

Search strategy of PubMed was as follows:

(1) ((Klatskin tumor) OR hilar cholangiocarcinoma) OR Perihilar Cholangiocarcinoma
(2) ((((liver transplantation) OR Liver Grafting) OR hepatic transplantation∗) OR “liver transplantations”) OR “liver transplant”
(3) Step 1 AND step 2

### 2.4 Study selection and data extraction

Two reviewers will select the included studies and extract relevant data independently from the studies. The selection process will be summarized according to PRISMA flow diagram. The data will include study characteristics, patients’ characteristics, data needed for quality assessment, and outcomes. Patients characteristics include type of inventions received, mean age, sex, sample, and tumor pathologic variables.

### 2.5 Risk of bias

The risk of bias will be independently evaluated by 2 investigators for each selected study using the Newcastle–Ottawa Quality Assessment Scale (NOS).^[8]^ Disagreements will be resolved by discussion another investigator. The scores of NOS range from 0 to 9, and scores >6 are considered as high quality.^[8]^

### 2.6 Data synthesis and statistical analysis

We will use STATA version 14.0 (College Station, TX) to perform the relevant statistical analysis. The statistical tests are 2-sided, and *P*-values <.05 are considered statistical significant. Pooled odds ratio will be calculated for dichotomous data.

The fixed-effects or random-effects model will be used to calculate the outcomes. In case of significant statistical heterogeneity, the random-effects model will be used. Heterogeneity of the included trials will be assessed by Cochran Q test and measured by the *I*^2^ statistic. Interpretation of the *I*^2^ values will be made by assigning attributes of low, moderate, and high in case of 0% to 25%, 25% to 50%, and above 75%, respectively.^[9]^ Statistical heterogeneity will be quantitatively evaluated by χ^2^ test with the significance set *P* < .10 or *I*^2^ > 50%.^[10]^ Begg funnel plot and Egger regression will be used to assess the publication bias.^[11,12]^

Sensitivity analysis will be performed to identify the stability of the result by omitting each of the enrolled studies or excluding low-quality studies.

## 3 Discussion

Currently, the optional surgical choice for HC is still uncertain. Both liver transplantation and surgical resection are available for selected patients with HC. Therefore, it is necessary for us to perform a high-quality systemic review and meta-analysis to investigate this question. We will conduct the meta-analysis to summarize all the current evidence and provide suggestions for clinical practice.

## Author contributions

Bo Zhang, Dan Cao and Zhaolun Cai conceived the concept and designed the study protocol. Zhaohui Cai and Zhou Zhao tested the feasibility of the study. Zhixin Chen, Chaoyong Shen, Yuan Yin, Xiaonan Yin, and Jian Wang wrote the manuscript. Bo Zhang, Dan Cao and Zhaolun Cai provided methodological advice, polished and revised the manuscript. All authors saw and approved the final version of the paper.

**Conceptualization:** Zhaolun Cai, Dan Cao, Bo Zhang.

**Investigation:** Zhaohui Cai, Zhou Zhao.

**Methodology:** Zhaolun Cai, Dan Cao, Bo Zhang.

**Writing – original draft:** Yuan Yin, Chaoyong Shen, Xiaonan Yin, Jian Wang, Zhixin Chen.

**Writing – review & editing:** Zhaolun Cai, Dan Cao, Bo Zhang.

## References

[1] Mansour JC Aloia TA Crane CH Hilar Cholangiocarcinoma: expert consensus statement. HPB (Oxford) 2015;17:691–9. 26172136 10.1111/hpb.12450 PMC4527854

[2] Taylorrobinson SD Foster GR Arora S Increase in primary liver cancer in the UK, 1979-94. Lancet 1997;350:1142–3. 10.1016/S0140-6736(05)63789-0 9343506

[3] Patel MDT Increasing incidence and mortality of primary intrahepatic cholangiocarcinoma in the United States. Hepatology 2001;33:1353–7. 11391522 10.1053/jhep.2001.25087

[4] Hong JC Jones CM Duffy JP Comparative analysis of resection and liver transplantation for intrahepatic and hilar cholangiocarcinoma: a 24-year experience in a single center. Arch Surg 2011;146:683–9. 21690444 10.1001/archsurg.2011.116

[5] Croome KP Rosen CB Heimbach JK Is liver transplantation appropriate for patients with potentially resectable de novo hilar cholangiocarcinoma? J Am Coll Surg 2015;221:130–9. 25872685 10.1016/j.jamcollsurg.2015.01.064

[6] Ethun CG Lopez-Aguiar AG Anderson DJ Transplantation versus resection for hilar cholangiocarcinoma: an argument for shifting treatment paradigms for resectable disease. Ann Surg 2018;267:797–805. 29064885 10.1097/SLA.0000000000002574 PMC6002861

[7] Liberati A Altman DG Tetzlaff J The PRISMA statement for reporting systematic reviews and meta-analyses of studies that evaluate health care interventions: explanation and elaboration. J Clin Epidemiol 2009;62:e1–34. 19631507 10.1016/j.jclinepi.2009.06.006

[8] Wells GA Shea BJ O’Connell D The Newcastle-Ottawa scale (NOS) for assessing the quality of non-randomized studies in meta-analysis. Applied Engineering in Agriculture 2014;18:727–34.

[9] Higgins JP Thompson SG Deeks JJ Measuring inconsistency in meta-analyses. Br Med J 2003;327:557–60. 12958120 10.1136/bmj.327.7414.557 PMC192859

[10] Gs HJP Cochrane handbook for systematic reviews of interventions version 5.1.0 (updated March 2011). Naunyn Schmiedebergs Arch Exp Pathol Pharmakol 2014;5:S38.

[11] Egger M Davey SG Schneider M Bias in meta-analysis detected by a simple, graphical test. BMJ 1997;315:629–34. 9310563 10.1136/bmj.315.7109.629 PMC2127453

[12] Begg CB Mazumdar M Operating characteristics of a rank correlation test for publication bias. Biometrics 1994;50:1088–101. 7786990

